# Quinone-Based Polymers for Label-Free and Reagentless Electrochemical Immunosensors: Application to Proteins, Antibodies and Pesticides Detection

**DOI:** 10.3390/bios3010058

**Published:** 2013-01-14

**Authors:** Benoit Piro, Steeve Reisberg, Guillaume Anquetin, Huynh-Thien Duc, Minh-Chau Pham

**Affiliations:** 1 Université Paris Diderot, Sorbonne Paris Cité, ITODYS, UMR 7086 CNRS, 15 rue J-A de Baïf, 75205 Paris Cedex 13, France; E-Mails: steeve.reisberg@univ-paris-diderot.fr (S.R.); guillaume.anquetin@univ-paris-diderot.fr (G.A.); mcpham@univ-paris-diderot.fr (M.-C.P.); 2 Université Paris XI, INSERM U-1014, Groupe Hospitalier Paul Brousse-94800 Villejuif, France; E-Mail: guy.duc@inserm.fr

**Keywords:** conducting polymer, square wave voltammetry, impedance spectroscopy, immunosensor, proteins, peptides, pollutants, electrochemical biosensor, label-free detection

## Abstract

Polyquinone derivatives are widely recognized in the literature for their remarkable properties, their biocompatibility, simple synthesis, and easy bio-functionalization. We have shown that polyquinones present very stable electroactivity in neutral aqueous medium within the cathodic potential domain avoiding side oxidation of interfering species. Besides, they can act as immobilized redox transducers for probing biomolecular interactions in sensors. Our group has been working on devices based on such modified electrodes with a view to applications for proteins, antibodies and organic pollutants using a reagentless label-free electrochemical immunosensor format. Herein, these developments are briefly reviewed and put into perspective.

## 1. General Introduction

### 1.1. Generalities

An antigen (Ag) is a nanometer-sized molecule with molecular weight higher than 1,500 Da to which the immune system of animals responds by synthesizing antibodies (Ab) able to selectively bind the antigen. There are five primary classes of antibodies, namely IgG, IgM, IgA, IgD and IgE, but only IgG antibodies are used in immunosensors. Ab-Ag binding is based on non-covalent interactions and is characterized by an association constant, K_a_, in the range 10^5^–10^13^ M. Polyclonal Ab are directed to different binding sites (epitopes) on Ag, with different affinities. Monoclonal Ab, specific to only one epitope of an Ag, are preferred. These Ab generally present higher affinity and specificity.

### 1.2. Immunoassays

**Figure 1:**
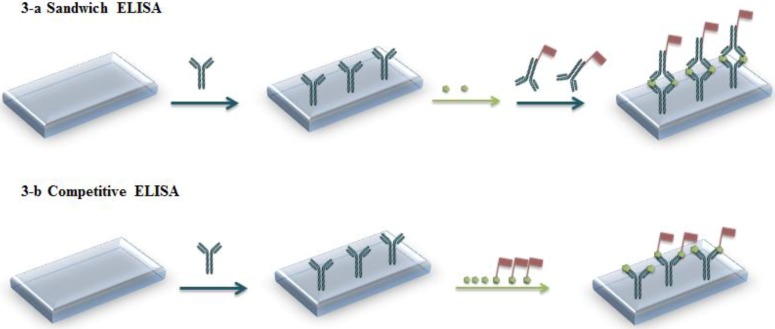
Principle of enzyme-linked immunosorbent assay (ELISA). (**a**) Sandwich format (**b**) Competitive format.

Analytical tools using Ab-Ag couples are called immunoassays. They are capable of direct and specific detection in complex samples, due to highly specific interactions between Ab and Ag. Commercially available immunoassay kits are inexpensive, simple, adapted to field use and constitute a rapid way of determining contaminants in environmental samples. Generally, the detection scheme relies on labeling with radioisotopes (radioimmunoassay, RIA, now scarcely used), chemiluminescent compounds (chemiluminescence immunoassay, CLIA) [1,2] or enzymes (enzyme immunoassay, EIA, and enzyme-linked immunosorbent assay, ELISA) [3,4,5,6]. Usually, the Ab or the Ag is immobilized on a substrate. In the most basic format, an immunocomplex (Ab-Ag) is formed upon contact with a solution containing the labeled analyte (generally the Ag). The analyte concentration in solution is then derived from that of bound analyte. In a more efficient sandwich format, Ab are immobilized on a substrate which is put in contact with the sample containing the Ag, then a labeled secondary antibody is added (see Figure 1(a)). Competitive formats also exist, in which competition takes place between non-labeled and labeled Ag for an immobilized Ab (Figure 1(b)). 

### 1.3. Immunosensors

Today, most immunoassays are performed using ELISA. They are generally performed in microtiter plates, which demand time and skilled operators. Moreover, ELISA tests are not efficient for simultaneous multiple analysis (multiplexing), continuous detection and transduction into an electronically usable signal. In the near future, biosensors using antibody-antigen complexes (called immunosensors) may provide faster and easier methods. An immunosensor consists of a sensing element (an immobilized Ab or Ag) and a transducer which translates the biomolecular recognition event into an electrical signal. This can be performed indirectly through SPR [7,8]. However, SPR is expensive, requires specialized technicians and is rarely employed for on-site measurement. In addition, optic-based detections in general are fragile. Transduction can also be performed through piezoelectric techniques [9] or near field microscopies (AFM, SECM) [10], with few of the same inconveniences. Field-effect transistors are competitive [11,12], but we focus in this paper on electrochemical sensors which are much more widely reported in the literature. 

### 1.4. Electrochemical Immunosensors

Electrochemical biosensors have attracted considerable attention due to the advantages of robustness, low cost, low power consumption, simplicity, high sensitivity, compatibility with mass manufacturing using existing micro-fabrication technologies, and portability; therefore they are excellent candidates for easy-to-use immunosensors. The most popular format uses a redox reaction from a labeled Ab (or Ag) which provides a current proportional to the analyte concentration. For example, Ab or Ag may be labeled with enzymes producing an electroactive product from a reagent added in the analytical solution [13]. Detection is generally performed by cyclic voltammetry, chronoamperometry or most frequently, impedimetry [14]. However, it would be more interesting to perform electrochemical transduction without labeling the biomolecules by a redox tag or adding redox reagent to the solution. This can be performed with conducting polymers (ECPs).

### 1.5. Electrochemical Immunosensors Based on Conducting Polymers

ECPs are smart materials because they present at the same time, grafting, coupling and transduction abilities [15,16,17,18] without help of a supplementary redox label put on the Ab or in solution. As Ab-Ag complexes are heavy and hindering, it is expected that their presence in the vicinity of the polymer/solution interface strongly influences ion diffusion from the solution to the polymer/solution interface and mixed charge transport inside the film. This typically leads to a measurable decrease in the electroactivity of the underlying conducting polymer. 

#### 1.5.1. Polypyrrole

The most popular ECP for biosensing applications is polypyrrole (PPy) [19,20,21]. In 1991, John *et al.* [22] described a method for incorporation of antibodies (anti-HSA) in PPy. It was shown by cyclic voltammetry that HSA (human serum albumin) interacted with the electrode surface while no response was obtained with pure PPy. Sadik and Wallace [23] optimized this method with detection limits in the order of nM [24]. Later, based on the same principle, an electrochemical immunosensor for direct detection of small organic antigenic pollutants like pesticides or antibiotics was developed. For example, polychlorinated biphenyls (PCBs) have been detected in water with a detection limit of 0.05 μg·L^−1^ [25,26,27,28]. 

#### 1.5.2. Other ECPs

Other conducting polymers were also used for detection of pollutants. For example, Shim *et al.* [29] fabricated a label free impedimetric immunosensor to detect bisphenol A (endocrine disrupting compounds released into the environment from many kinds of polycarbonate plastics, epoxy resins of food cans, *etc.*) using polythiophene, with a detection limit of 0.3 ± 0.07 ng·mL^−1^. Khan *et al.* [30] performed an impedimetric immunosensor based on a chitosan/polyaniline hybrid to detect ochratoxin-A (a mycotoxin found in food products, human blood, breast milk, tissues and organs of animals). Other polymers were also used, and more particularly polyquinones, which present particular redox properties. For example, a series of aminonaphthoquinones and aminoanthraquinones were originally developed in the 80’s and 90’s for electrocatalytic purposes or energy storage [31,32,33,34,35,36,37]. More recently, other polyquinone films were developed to be used as transducers in biosensors [38,39]. Indeed, even if polyquinone derivatives have been much less investigated than other ECPs, they present good biocompatibility, easy bio-functionalization and remarkably stable electroactivity in neutral aqueous medium [40]. These properties can be used to probe biomolecular interactions [41,42,43,44] due to the high sensitivity of the quinone group to changes in its local physico-chemical environment [45,46,47]. 

## 2. Recent Advances on Polyquinone-Modified Electrodes for Immunosensing

### 2.1. General Approach

#### 2.1.1. Principles

**Figure 2:**
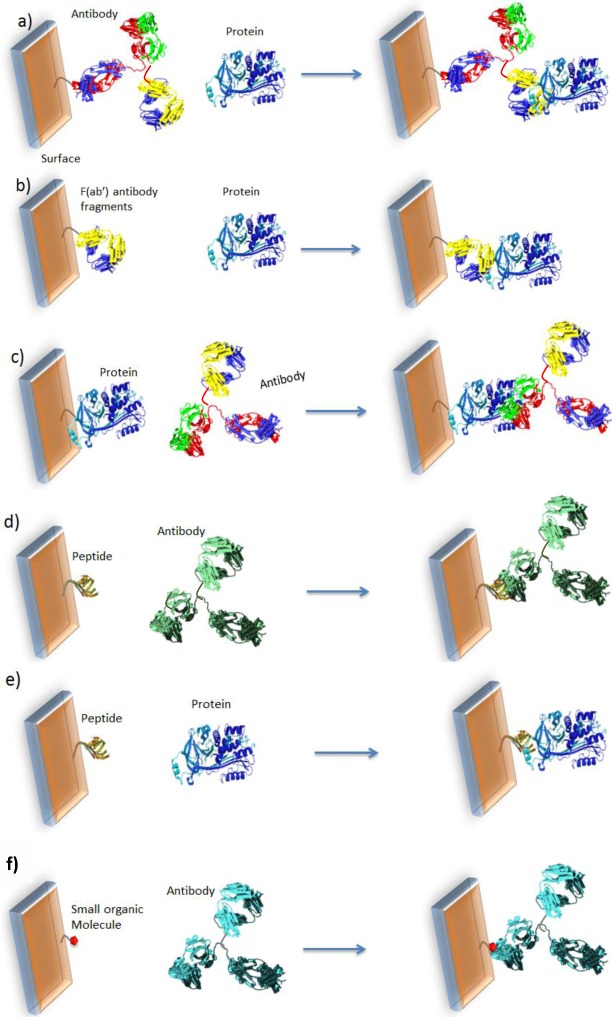
Classical formats used for detections of proteins or antibodies. (**a**) Grafted antibodies (Ab) to detect proteins; (**b**) Grafted antibody fragment F(ab’) to detect proteins; (**c**) Grafted protein to detect Ab. Use of peptides to detect (**d**) antibodies or (**e**) proteins. (**f**) Use of small organic molecules.

The major bottleneck is how to achieve direct electrochemical transduction when there is no intrinsic charge transfer reaction following molecular recognition. The most original and innovative idea is to directly immobilize the redox transducer on the sensor surface so that its electroactivity can be influenced by steric hindrance of heavy molecules (Ab or proteins) in its neighborhood. The detection of the target is performed simply by recording the redox current before and after recognition. This approach allows the development of easy-to-use, reagentless and label-free electrochemical devices. Several sensing architectures could be designed for such an approach, schematized and summarized in Figure 2 below.

Cases a–c, Figure 2, describe the most common approaches, which use relatively bulky probes. In order to reduce the size of the grafted probe, it is also possible to use peptides, as shown for cases d,e. Finally, small organic molecules may be used (f) instead of proteins or peptides [48].

#### 2.1.2. Design

**Figure 3:**
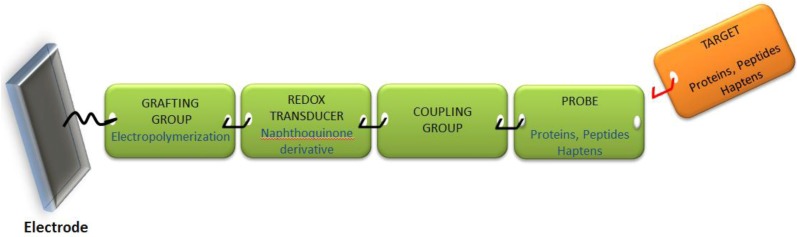
Schematic view of the “elemental bricks” needed to construct a versatile reagentless and label-free electrochemical sensor.

Because the sensor’s architecture must be adaptable to any format from among those schematized in Figure 2, it has to be engineered from “elemental bricks” joined together to form the whole electrochemical sensor, namely the grafting group, the redox transducer and the probe able to selectively complex the target molecule. This construction is schematized in Figure 3 below. 

We selected electropolymerization as the strategy to graft the sensing material, using hydroxynaphthoquinone monomers, which polymerize by electrooxidation of the hydroxyl group. The quinone plays the role of redox transducer, and we developed a coupling strategy to directly graft a spacer (e.g., an alkyl chain bearing a functional group) on the α-carbon of the quinone. This spacer is then used to couple the probe (antibody, protein, peptide or modified hapten).

**Figure 4:**
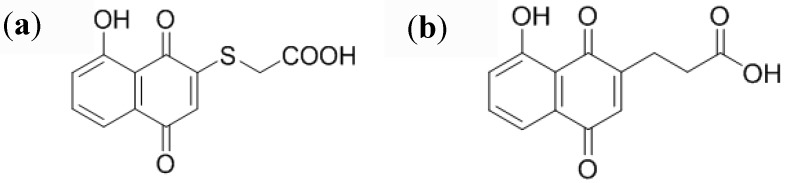
(**a**) Structure of 5-hydroxy-2-thioacetic acid-1,4-naphthoquinone) (HSNQA) and (**b**) 5-hydroxy-1,4-naphthoquinone-3-propionic acid (HNQA).

We first synthesized 5-hydroxy-2-thioacetic acid-1,4-naphthoquinone.(HSNQA) (Figure 4(a)). The reaction of thiols with various hydroxynaphthoquinone derivatives leads, in one step, to substituted quinone rings, under mild conditions [49]. Another spacer was also designed by straightforward carbon-carbon coupling, leading to 5-hydroxy-1,4-naphthoquinone-3-propionic acid (HNQA) (Figure 4(b)) [42].

We obtained a multifunctional conjugated copolymer poly(5-hydroxy-1,4-naphthoquinone-co-(5-hydroxy-2-thioacetic acid-1,4-naphthoquinone), poly(HNQ-co-HSNQA) and used it as the immobilizing and transducing element for a label-free electrochemical immunosensor [44]. Biomolecules can be coupled through peptide links between the –COOH group and the terminal –NH_2 _group on the bioprobe. The quinone group was used for its redox properties. It is well known that the quinone/hydroquinone system presents an electroactivity which is sensitive to its local environment, particularly cations, protons or sodium ions in aqueous solution. The redox reaction in the film involves the reaction Q + 2H^+^ + 2e^−^ ⇌ QH_2_ or Q + 2Na^+^ + 2e^−^ ⇌ Q^2−^, 2Na^+^. Presence of heavy biomolecules in the vicinity of the quinone group leads to a local modification of the proton and sodium apparent diffusion coefficient; therefore quinone electroactivity changes [38].

### 2.2. Applications

#### 2.2.1. Proteins as Probes

**Figure 5:**
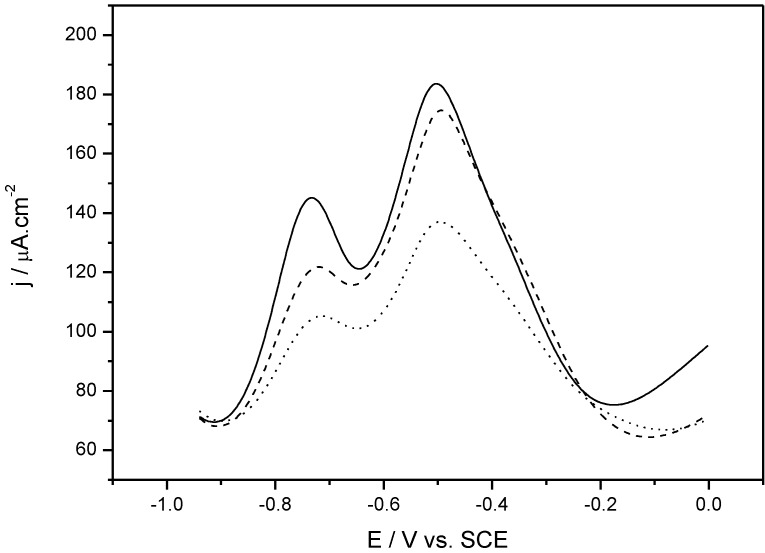
Square wave voltammetry (SWV) obtained for poly(HNQ-co-HSNQA)-modified electrodes before ovalbumin (OVA) grafting (solid line), after OVA grafting (dashed line) and after anti-ovalbumin (αOVA) complexation (dotted line). Serum dilution: 1/50 [44].

Proteins are macromolecules made of α-amino acids linked together by peptide bonds. The molecular weight is a fundamental characteristic of each protein; it is generally greater than 10,000 Da (10 kDa). The typical model used in immunosensors for the study of protein/antibody interactions is the ovalbumin/anti-ovalbumin (OVA/αOVA) system. We used this system as a model in our quinone-based biosensor. Poly(5-hydroxy-1,4-naphthoquinone-co-5-hydroxy-2-thioacetic acid-1,4-naphthoquinone) (poly(HNQ-co-HSNQA)), was used as both the immobilization and transduction element. OVA was grafted on this polymer and used as probe to detect αOVA present in solution. The immune complex was detected by recording the electrochemical signal using square wave voltammetry (SWV, Figure 5). In aqueous solution, quinones transfer two electrons and two protons in a concerted process. We assume that the presence of two pairs of peaks in PBS is due to two different types of quinones on the electrode surface: in the bulk of the polymer and at the polymer/electrolyte interface. The current decreased upon addition of αOVA, whereas no signal was observed upon addition of non-specific anti-KLH or diluted (1/50) normal serum. 

**Figure 6:**
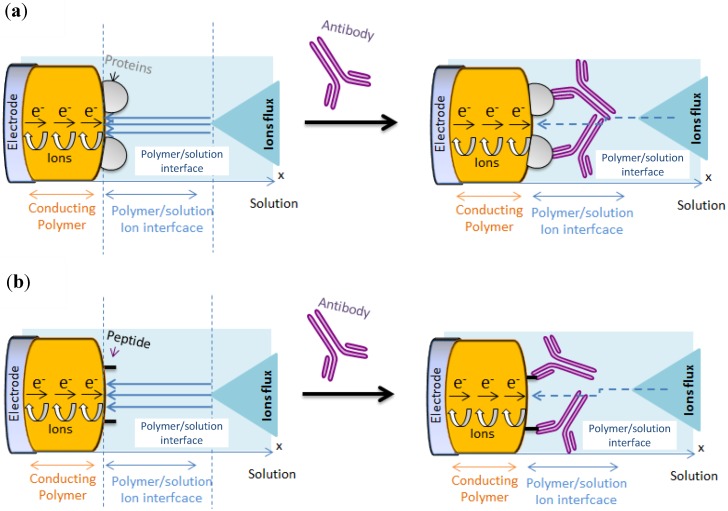
Transduction principle for poly(HNQ-co-HSNQA)/OVA/αOVA sensor (**a**) case of protein/antibody complex ; (**b**) peptide/antibody complex.

It was proposed (Figure 6(a)) that the lowering of the redox activity of the quinone groups embedded in the polymer film is due to the steric hindrance of αOVA (150 kDa) which is barely four times bigger than OVA (45 kDa) [44]. 

In the configuration of case a, the sensitivity of the sensor is limited by the fact that the probe (the protein) is relatively big. In terms of mean area occupied by proteins on the electrode surface, we can take the power 2/3 of the molecular weight ratio to determine the relative projected area between probe and target on the electrode surface. It gives (150/45)^2/3^ = 2.2, which means that αOVA is only ca. two times more hindering than OVA; This is low and not optimal. One way to solve the problem is to reduce the probe size, e.g., using peptides instead of whole proteins (Figure 6(b)).

#### 2.2.2. Peptides for Biomolecule Grafting

**Figure 7:**
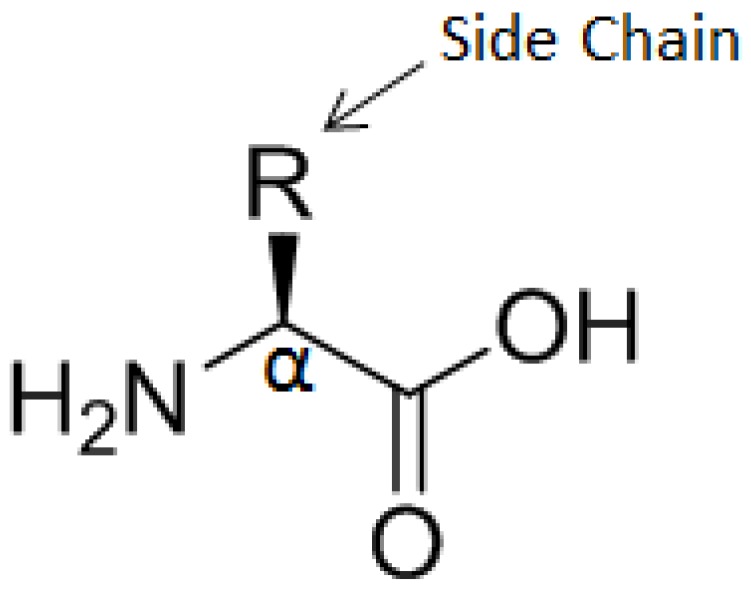
General structure of α-amino acids.

α-Amino acids are the building blocks of proteins and peptides. They carry a primary amine group bound to the α-carbon of the carboxylic acid group, which gives them the generic structure H_2_N-CHR-COOH wherein R represents the side chain (a functional group) which identifies the α-amino acid. Their general structure is shown in Figure 7. 

**Figure 8:**
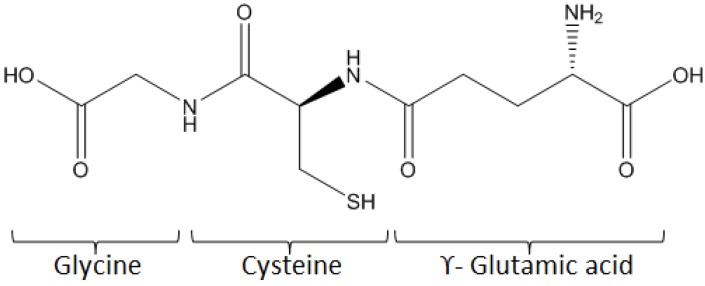
Structure of glutathione.

The α-Amino acids can bind to each other through an amide bond between the α-carboxyl group of one and the α-amine group of another, to form peptides. Compared to proteins, peptides are smaller, structurally less complex and more stable. Their synthesis can be automated, which is a considerable advantage compared to proteins and allows new biological and biomedical applications, as well as for diagnostics. Among other peptides of interest, glutathione (γ-L-glutamyl-L-cysteinyl-glycine) is potentially an efficient linker for biomolecule immobilization on the surface. Indeed, it is a tripeptide, bearing one primary amine, two amide groups, two carboxylic groups and one thiol group, all these groups being chemically reactive (see Figure 8). 

**Figure 9:**
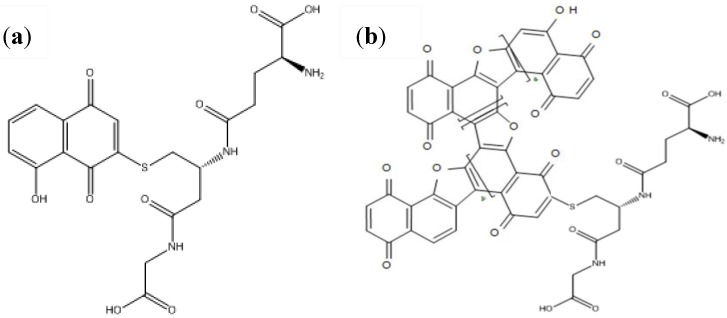
(**a**) Structure of the juglone-gluthathione conjugate (HNQ-Glu). (**b**) Structure of the corresponding conducting polymer poly(HNQ-Glu).

We recently developed direct grafting of glutathione (using the thiol group present on the side chain of cysteine) onto 5-hydroxy-1,4-naphthoquinone (HNQ) [41] to obtain 5-hydroxy-3-γ-L-glutamyl-L-cysteinyl-glycine-1,4-naphthoquinone (HNQ-Glu, Figure 9(a)) which was electropolymerized to give the polymer structure shown in Figure 9(b).

The glutathione moiety was used as the anchoring group for oligonucleotide immobilization in a DNA sensor [41].

#### 2.2.3. Peptides as Probes

Protein-protein or antigen-antibody interactions involve limited areas of both partners. Activities and biological functions of proteins can be analyzed by studying the interactions between shorter synthetic peptide fragments constituting the active site of a protein and their ligands, e.g., for the study of interactions between specific peptide epitopes of a protein and its specific antibody. Following this idea, we developed a strategy for the detection of Human papillomaviruses (HPVs). 

It is known that genotypes 16 and 18 of Human papillomavirus (HPV-16 and HPV-18) are implicated in cervical cancer [50,51]. During the natural course of HPV infection, the presence of antibodies is usually observed [52]. Until now, most serological analyses have relied on enzyme-linked immunosorbent assays (ELISAs) [53] or antibody displacement assays [54]. Electrochemical biosensors for HPV detection are scarce. The most common approach consists in using immobilized antibodies which target the virus. A smarter approach consists in immobilizing a peptide present on the virus capside which can target antibodies in the infected serum [55,56]. Electrochemical detection of antibodies using peptide probes have been proposed, e.g., for the *Nile* virus [57]. For HPV detection, we proposed to target antibodies using HPV-16-L1 peptide (311–325 sequence, Asn-Leu-Ala-Ser-Ser-Asn-Tyr-Phe-Pro-Thr-Pro-Ser-Gly-Ser-Met), allowing HPV detection without labeling or adding reagent into solution. HPV-16-L1 (1,8 kDa) was grafted on the electrode to probe the HPV-16 antibody (much bigger, ca. 150 kDa) [42]. In this format, the big difference in size makes transduction of the molecular recognition much more sensitive than for classical ELISA-like architectures, the relative projected area between probe and target being (150/1.8)^2/3^ = 19 >>2.2 (see end of Section 2.2.1). 

**Figure 10:**
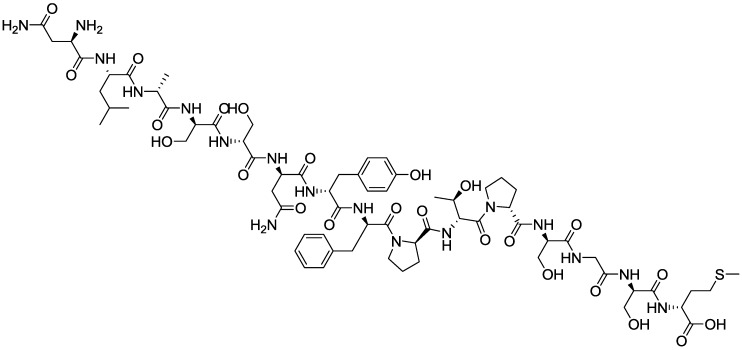
Structure of Human papillomavirus (HPV-16-L1).

**Figure 11:**
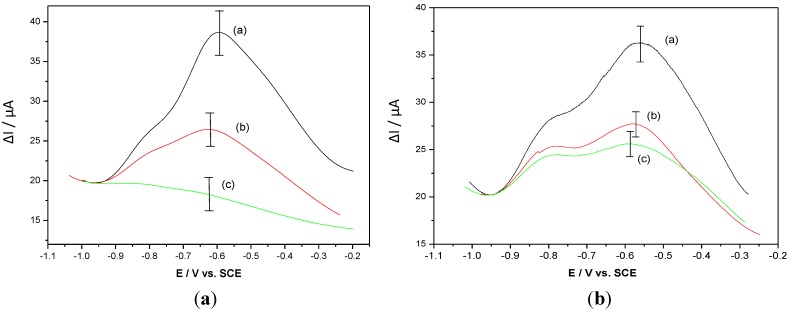
(**a**) SWV of a poly(HNQ-co-HSNQA) recorded in PBS before grafting (curve a), after grafting of 5 × 10^−8^ M HPV-16-L1 (curve b) and after complexation with 5 × 10^−8^ M α-HPV (curve c). (**b**) SWV of a poly(HNQ-co-HSNQA) recorded in PBS before grafting (curve a), after grafting of 5 × 10^−8^ M HPV-16-L1 (curve b) and after complexation with 5 × 10^−8^ M α-OVA (curve c) [42].

SWV was used to characterize the presence of the antibody. As expected, formation of the HPV-16-L1/anti-HPV complex induces a strong decrease in the polymer electroactivity (Figure 11(a)). If the same experiment is performed with a non-target antibody (e.g., anti-OVA), the current drop is lower than for anti-HPV (Figure 11(b)), which demonstrates the specificity of the molecular recognition. 

**Figure 12:**
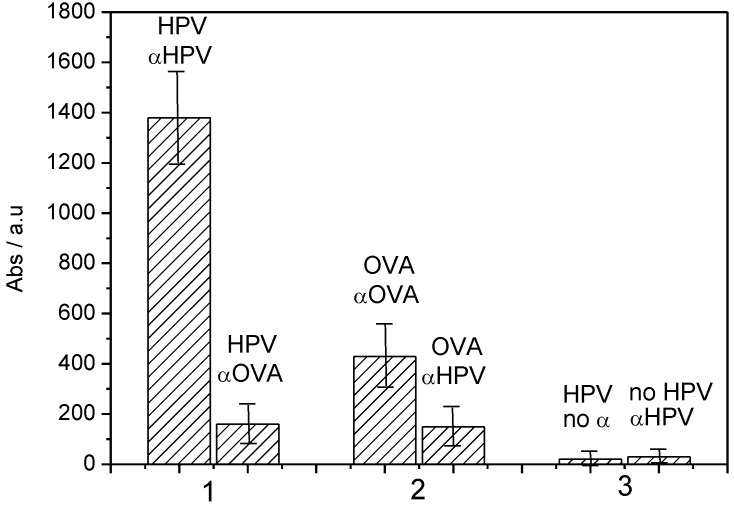
Absorbance obtained for spectrophotometric ELISA assays corresponding to: (**1**), HPV-16-L1 grafted, + αHPV (left bar) or + αOVA (right bar). (**2**) OVA grafted, + αOVA (left bar) or + αHPV (right bar). (**3**) HPV grafted + no antibody (pure PBS) (left bar), or nothing grafted + αHPV (right bar) [42].

These results were confirmed by traditional ELISA. It is important to note that due to the chemical nature of the quinone group and its redox process (the film being neutral or negatively charged, but never positively charged), poly(HNQ-co-HSNQA) enables the avoidance of non-specific adsorption of proteins on its surface (Figure 12) and therefore avoids false positives.

This work demonstrates the interest in replacing the heavy Ag probe (in this case, HPV virus) by much smaller fragments such as an oligopeptide constituted of 10 to 20 amino acids, which presents a much smaller size than a bulky (and dangerous to handle) virus. 

#### 2.2.4. Small Organic Molecules as Probes

Analysis of potable water is correlated with public health and its regulation is under development. More than 10,000 molecules are liable to be regulated within the forthcoming years. For metrology of micro-pollutants in water (for trace quantities of the order of micrograms per liter), analyses are currently mainly achieved through chromatographic techniques coupled to mass spectrometry. However, to gain time and cost we must avoid the separation step. To keep specificity without separation, antibodies are particularly efficient and have already been used, e.g., to detect pesticides [58,59]. For this application, most of the label-free immunosensors described in the literature rely on a transduction format based on impedance [60,61] or amperometry [62] using a redox label. However, it has been shown that the introduction of appropriate functionalities through chemical modification of the sensor surface can provide good sensing abilities without the help of a label [18]. 

Atrazine (6-chloro-*N*-ethyl-*N*-[1-methylethyl]-1,3,5-triazine-2,4-diamine; ATZ) is a widely used pesticide that constitutes an excellent model for small organic pollutants for which antibodies exist. One of the most recent and pertinent examples has been reported by Cosnier *et al.* [63] who have shown that it is possible to detect 10 pg·mL^−1^ atrazine by Electrochemical Impedance Spectroscopy (EIS) using an immunosensor based on polypyrrole film N-substituted by nitrilotriacetic acid electrogenerated on gold electrodes. An excellent overview of detection systems for atrazine is given in [64].

It is noteworthy to recall the ability of an antibody to react with more than one antigen. This is known as cross-reactivity and arises because the cross-reacting antigen shares a structure similar to that of the immunizing antigen used to generate antibodies. We used this ability to develop a novel approach and applied this to atrazine (ATZ) detection. It is based on the competitive complexation between the anti-atrazine (αATZ) antibody on one side and atrazine present in the analyzed sample or hydroxyatrazine (HATZ) immobilized on the sensor surface on the other side, in a true direct and label-free format (no redox probe added in solution). In this particular case, the antibody αATZ generated from ATZ can also bind to HATZ (the hydroxylated atrazine), but with lower affinity [65,66,67].

**Figure 13:**
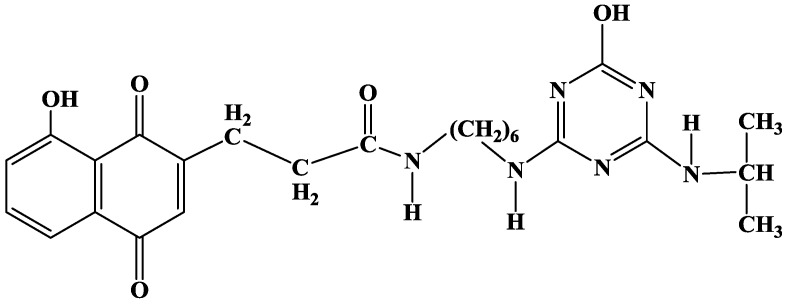
JUG-HATZ structure.

We recently developed [47,68] an electrogenerated polyquinone film functionalized by a hydroxyatrazine moiety, N-(6-(4-hydroxy-6-isopropylamino-1,3,5-triazin-2-ylamino)hexyl)-5-hydroxy-1,4-naphthoquinone-3-propionamide (JUG-HATZ), which contains three functional groups: the hydroxyl group for subsequent electropolymerization, the quinone group to be used as redox transducer and hydroxyatrazine as bioreceptor element (Figure 13). 

**Figure 14:**
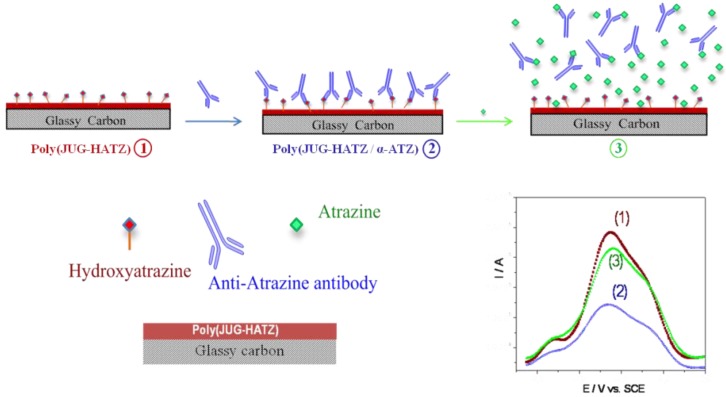
Immunosensor principle based on the cross-reactivity of an antibody. Example of atrazine (ATZ) detection [47].

After formation of the HATZ/αATZ complex, the faradic current of the quinone group decreases (Figure 14, step 2). The electrode modified by poly(JUG-HATZ) where αATZ is complexed is then utilized to detect ATZ in solution. Indeed, αATZ preferentially binds to ATZ, so that a displacement equilibrium occurs (Figure 14, step 3) between ATZ in solution and HATZ incorporated in the polymer. Addition of free atrazine removes the complexed antibodies from the electrode surface leading to an increase in electroactivity. It is proposed that the removal of the αATZ enhances the ionic flux through the interface and leads to this current increase. Here, the relative projected area between probe and target is (150/0.3)^2/3^ = 63, even higher than for peptide probes (see Section 2.2.3), which may explain the high sensitivity of this architecture. 

**Figure 15:**
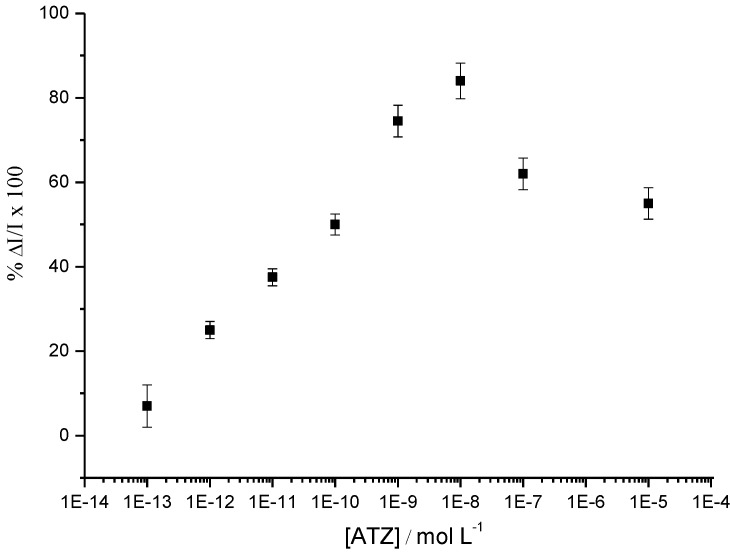
Percentage of ΔI/I measured by SWV at −0.45/SCE, after addition of ATZ for concentrations from 1 pM up to 10 µM. ΔI = I_poly(JUG-HATZ)_ – I_poly(JUG-HATZ/α-ATZ)_ and I = I_poly(JUG-HATZ/α-ATZ)_ [47].

This *signal-on* system allows detection of one of the lowest atrazine concentrations (1 pM) reported in the literature for this kind of reagentless and label-free electrochemical immunosensor (Figure 15).

**Figure 16:**
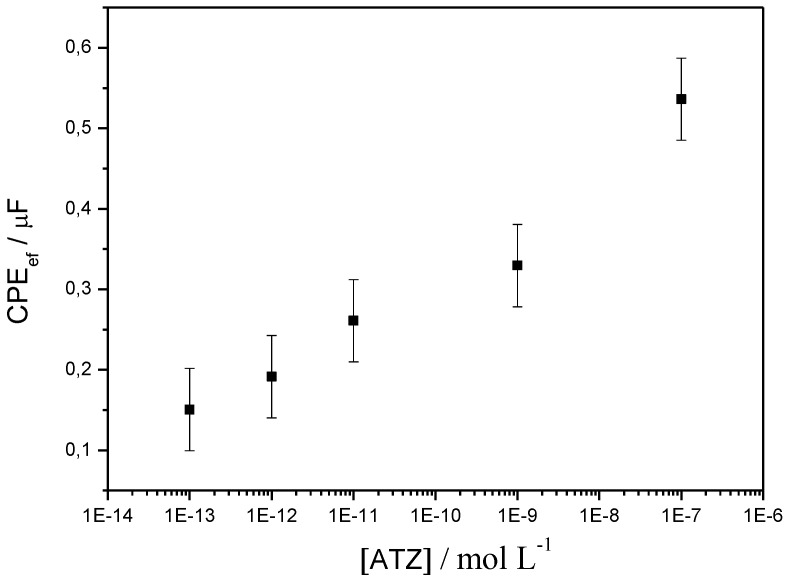
*CPE_ef_* as a function of ATZ concentration, from electrochemical impedance spectroscopy (EIS) experiments, on a poly(JUG-HATZ/α-ATZ)-modified electrode. Results obtained for an offset potential of −0.5 V *vs*. SCE [68].

Similar experiments were performed using electrochemical impedance spectroscopy (EIS). The process allows to detect atrazine with low detection limit (0.2 ng·L^−1^) in a true label-free format (no redox probe added in solution) by following changes in the electrochemical impedance of the sensor. αATZ has a molecular weight of ca. 150 kDa and an hydrodynamic volume of about 25 nm^3^, which makes a projected surface of 25^2/3^ = 8.5 nm^2^. These dimensions are able to significantly decrease the electrolyte-film interface capacitance. On the contrary, when the antibody is removed, *CPE_ef_* increases, as illustrated by the results shown on Figure 16.

The limit of detection can be estimated at 10^−12^ M, ca. 0.2 ng·L^−1^. Variations are relatively linear (on a log scale) up to 10^−7^ M (ca. 20 mg·L^−1^), which covers concentrations found in real ground water samples. 

This approach is versatile: it can be extended to numerous other targets (pesticides, organic pollutants or toxins) providing they are antigenic. Work is in progress with such molecules, e.g., bisphenol A, ochratoxin A, isoproturon and glyphosates.

## 3. Conclusions

The possibility to detect very small amounts of proteins, peptides or pollutants using a cheap technique offers numerous opportunities for point-of-care diagnostics and on-site monitoring. Electrochemical devices transform the analyte capture into an electrical signal and benefit from the expertise developed in the context of microelectronics. This allows consideration of mass production and miniaturization at low cost. The vast majority of existing systems are based on indirect detection by amperometry and need either to label targets by a redox molecule or an enzyme, or to add a redox marker in solution. The need to add a reagent is a drawback which was recently bypassed by the development of direct, reagentless and label-free methods, either using amperometry or impedance. The use of electroactive layers immobilized on electrodes, e.g., ECPs, is promising. As these direct methods are mainly based on steric effects, special attention must be paid to the probe and target dimensions. Typical ELISA-based methods, for which the probe is bigger than the target, are not ideal. It is therefore crucial to decrease the probe size, using Fab’_2 _antibodies, light proteins or even peptides and small haptens. 

## Appendx

### Experimentals

Phosphate buffer saline (PBS) had a pH of 7.4 and was provided by Sigma. Aqueous solutions were made with ultrapure (18 MΩ cm) water. Glassy carbon electrodes (GC, 3 mm in diameter, S = 0.07 cm^2^) were purchased from BASInc. 5-hydroxy-1,4-naphthoquinone (JUG), 1-naphthol (1-NAP), lithium perchlorate (LiClO_4_), acetonitrile (ACN) and ethanol (EtOH), practical grade, were from Sigma-Aldrich. Atrazine (ATZ) and atrazine-desethyl-2-hydroxy (ATD) were purchased from Supelco (USA). Anti-atrazine (α-ATZ) antibody (monoclonal) was purchased from Thermo Scientific, USA. A. OVA (egg albumin, 2× crystallized) was purchased from Calbiochem, La Jolla, CA, USA. HPV-16-L1 peptide (311–325 sequence, *i.e.*, Asn-Leu-Ala-Ser-Ser-Asn-Tyr-Phe-Pro-Thr-Pro-Ser-Gly-Ser-Met) was purchased from Genscript, USA. Secondary Goat F(ab’)_2_ anti-Mouse Ig conjugated to horseradish peroxidase was purchased from Tebu (Le Perray-en-Yvelines, France). Antibodies against HPV (αHPV, mouse anti-papillomavirus 16 L1 late protein, monoclonal antibody) and against OVA (αOVA) were obtained from AbD serotec (Morphosys, UK). The electrochemical synthesis of the polymer films was carried out by electro-oxidation of 5 × 10^−3^ M JUG-HATZ + 10^−3^ M 1-NAP + 0.1 M LiClO_4_ in ACN on GC electrodes, under Ar, at a constant potential of 1.25 V (*vs*. SCE) during 600 s. Square wave voltammetry (SWV) was used to characterize α-ATZ complexation and ATZ detection. The following parameters were used: pulse height 50 mV, pulse width 50 ms, scan increment 2 mV, frequency 12.5 Hz. EIS was performed using an Autolab PGSTAT30 equipped with the FRA module. Impedance spectra were recorded in PBS buffer at room temperature at a given offset potential within a frequency range from 10 kHz to 100 mHz with a perturbation amplitude of 10 mV. As a pretreatment before each experiment, a constant potential corresponding to the one used for EIS was imposed, for 120 s. Solutions were systematically deaerated with Ar before and during experiments.
